# Differential effect of angiotensin II and blood pressure on hippocampal inflammation in mice

**DOI:** 10.1186/s12974-018-1090-z

**Published:** 2018-02-28

**Authors:** M. Florencia Iulita, Diane Vallerand, Mélissa Beauvillier, Nathalie Haupert, Corinne A. Ulysse, Audrey Gagné, Nathalie Vernoux, Sonia Duchemin, Michaël Boily, Marie-Ève Tremblay, Hélène Girouard

**Affiliations:** 1 Department of Neurosciences, Université de Montréal, 2960 Chemin de la Tour, Montréal, Québec H3T 1J4 Canada; 2 Groupe de recherche sur le système nerveux central (GRSNC), Université de Montréal, 2960 Chemin de la Tour, Montréal, Québec H3T 1J4 Canada; 3 Department of Pharmacology and Physiology, Université de Montréal, Pavillon Roger-Gaudry, 2900 Boulevard Édouard-Montpetit, Montréal, Québec H3T 1J4 Canada; 4 Axe Neurosciences, CRCHU de Québec-Université Laval, 2705 Boulevard Laurier, Québec, Québec G1V 4G2 Canada; 5 Department of Molecular Medicine, Université Laval, 1050, Avenue de la Médecine, Québec, Québec G1V 0A6 Canada; 6 Centre de recherche de l’Institut universitaire de gériatrie de Montréal, 545 Queen Mary Rd, Montréal, Québec H3W 1W6 Canada

**Keywords:** Angiotensin II, Hypertension, Inflammation, Blood pressure, Microglia, TNF-α, GFAP, Iba-1, Hydralazine

## Abstract

**Background:**

Angiotensin II (Ang II), a peptide hormone involved in the development of hypertension, causes systemic and cerebral inflammation, affecting brain regions important for blood pressure control. The cause-and-effect relationship between hypertension and inflammation is two-way, but the role of blood pressure in the induction of cerebral inflammation is less clear. The vulnerability of specific brain regions, particularly those important for memory, is also of interest.

**Methods:**

We used molecular biology approaches, immunohistochemistry, and electron microscopy to examine the interdependence between the hypertensive and pro-inflammatory effects of Ang II. We examined the effect of blood pressure by administering a subpressive (200 ng/kg/min) or a pressive Ang II dose (1000 or 1900 ng/kg/min) with and without hydralazine (150 mg/L) for 1 week and used phenylephrine to increase blood pressure independently of the renin-angiotensin system.

**Results:**

Ang II increased ionized calcium-binding adaptor molecule 1 (Iba-1) levels (marker of microgliosis) in the whole brain and in the hippocampus in a dose-dependent manner. Pressive Ang II induced specific changes in microglial morphology, indicating differences in functional phenotype. An increase in hippocampal glial fibrillary acidic protein (GFAP) was seen in mice receiving pressive Ang II, while no induction of cerebral gliosis was observed after 7 days of subpressive Ang II infusion. Although phenylephrine led to increased astrogliosis, it did not affect Iba-1 expression. Pressive Ang II stimulated TNF-α production in the hippocampus, and daily treatment with hydralazine prevented this increase. Hydralazine also reduced GFAP and Iba-1 levels. With longer perfusion (14 days), subpressive Ang II led to some but not all the inflammatory changes detected with the pressive doses, mainly an increase in CD68 and Iba-1 but not of GFAP or TNF-α.

**Conclusions:**

Blood pressure and Ang II differentially contribute to hippocampal inflammation in mice. Control of blood pressure and Ang II levels should prevent or reduce brain inflammation and therefore brain dysfunctions associated with hypertension.

**Electronic supplementary material:**

The online version of this article (10.1186/s12974-018-1090-z) contains supplementary material, which is available to authorized users.

## Background

Hypertension is a serious public health issue, being highly prevalent in the elderly [1] and a leading risk factor for stroke [2], cognitive impairment, and dementia [3]. Despite the vast array of drugs to lower blood pressure, hypertension control remains suboptimal stressing the need for better therapeutic management.

Besides reflecting an increase in arterial blood pressure, hypertension consists of a chronic, low-grade inflammation involving innate and adaptive immune cells [4]. The link between systemic inflammation and high blood pressure has been well described by human [5–7] and animal data [8–14]. However, the effects of hypertension on the pathogenesis of cerebral inflammation are less understood.

Studies examining the role of cerebral inflammation in experimental hypertension have focused on the paraventricular nucleus (PVN), a region of the hypothalamus involved in the sympathetic control of blood pressure [15]. Using anti-inflammatory approaches (with minocycline or IL-10 administered directly to the brain) or depleting microglia by intra-cerebro-ventricular administration of diphtheria toxin (DT) to transgenic CD11b-DT receptor mice, these studies have shown that inflammation and microglia play a central role in the development of high blood pressure in mice and rats [16, 17]. While finding that inflammation influenced blood pressure, they also showed that hypertension, which was achieved via systemic infusion of angiotensin II (Ang II), was capable of producing increases in microglial density, soma enlargement, and reductions in microglial process length, as well as greater production of pro-inflammatory cytokines (IL-1β, IL-6, and TNF-α) in this brain region [16–18].

Given the strong associations between mid-life hypertension and late-life dementia [19–21], the involvement of other vulnerable brain structures such as the hippocampus, a key region involved in memory formation, is also of significant interest. Likewise, whether the cerebral inflammatory effects of circulating Ang II are a consequence of its own cellular actions only, whether they are mediated by its increase in blood pressure, or whether blood pressure itself can do so independently of Ang II remains unclear.

Therefore, this study was set to answer these questions. We administered Ang II in the systemic circulation of C57BL/6 mice during 7 or 14 days and examined the inflammatory response in the whole brain and in the hippocampus using a multidisciplinary approach. In order to dissect the effects of Ang II and blood pressure, we delivered pressive versus subpressive Ang II doses and phenylephrine, or used the peripheral vasodilator hydralazine to prevent hypertension. Our results show that Ang II and blood pressure can differentially contribute to the development of hippocampal inflammation in mice.

## Methods

### Animals

Ten to 12-week-old male C57BL/6 mice (Charles River Laboratories, Saint-Constant, Canada) were housed individually in a temperature-controlled room with a 12-h light-dark cycle and access to food and water ad libitum. Following acclimatization, mice were randomly assigned to experimental groups. The precise number of animals used for each experiment is specified in the Results section and in the figure legends.

### Osmotic minipump implantation and hydralazine treatment

Alzet osmotic minipumps (model 1007D, Durect Corporation, Cupertino, USA) were subcutaneously implanted as previously described [22]. Mice received bupivacaine hydrochloride (Marcaine, CDMV, Canada, 2 mg/kg s.c.) at the site of the incision before and after minipump implantation. Each pump delivered 200 or 1000 ng/kg/min of Ang II (Millipore-Sigma, Maine, USA) at a rate of 0.5 μL/h during 7 days. Saline (0.9%) was used for the control groups. For ionized calcium-binding adaptor molecule 1 (Iba-1) and glial fibrillary acidic protein (GFAP) analysis, an additional group of mice was included receiving either Ang II 1900 ng/kg/min or phenylephrine (19 μg/kg/min). Phenylephrine (Sigma-Aldrich, Oakville, Canada) was infused via osmotic minipumps, in the same conditions. A separate group of mice was implanted with minipumps that delivered the subpressive Ang II dose during 14 days (model 1002, rate 0.25 μL/h, Durect Corporation, Cupertino, USA). For hydralazine treatment (Hyd), mice were divided into four groups: mice receiving regular drinking water (control and Ang II 1000 ng/kg/min) and mice receiving 150 mg/L of hydralazine hydrochloride (Sigma-Aldrich, Oakville, Canada) daily in the drinking water (control + Hyd and Ang II + Hyd groups). Hydralazine treatment started 3 days before the minipump implantation and continued during the 7 days of Ang II perfusion. The hydralazine solution was replaced each day. The optimal concentration of hydralazine was chosen based on a pilot study. For a positive control of systemic inflammation, mice were intraperitoneally (i.p.) injected with a single dose of lipopolysaccharides (LPS from *Escherichia coli*, 2 mg/kg, Sigma-Aldrich, Oakville, Canada) and sacrificed 3 h later.

### Blood pressure measurement

Blood pressure was monitored by non-invasive tail-cuff plethysmography (Kent Scientific Corporation, Torrington, USA). Blood pressure assessment began 3 days before minipump implantation in order to habituate the mice to the procedure and continued at three different intervals (days 0, 4, and 7) during 1 week. Mice were placed on a heating platform for 10 min before assessment of blood pressure. A minimum of five measurements were taken until the blood pressure stabilized and, following stabilization, a minimum of eight measurements were taken per mouse and averaged for analysis. Blood pressure was monitored by the same person at the same time of the day.

### Western blotting

Equal amounts of proteins from whole brain homogenates (75 μg) were subjected to electrophoresis and transferred to nitrocellulose membranes (BioRad, ON, Canada). Membranes were probed with rabbit anti-Iba-1 (1:500, Wako Inc., Richmond, USA) or mouse anti-GFAP (1:2000, Millipore-Sigma, Maine, USA) antibodies overnight at 4 °C. Rabbit anti-pan actin (1:1000, New England Biolabs, Ltd., Whitby, Canada) was used as loading control. Densitometric analysis was performed using ImageJ software (National Institutes of Health, Bethesda, USA), and the ratio Iba-1/actin or GFAP/actin was expressed relative to the control group. Details on tissue preparation and additional details on the Western blot protocol are available in Additional file 1: Supplementary Methods.

### RNA extraction and quantitative real-time PCR (qRT-PCR)

Details on tissue preparation are available in Additional file 1: Supplementary Methods. Hippocampal RNA was extracted with the RNeasy Plus kit (Qiagen, Toronto, Canada) and retrotranscribed with the qScript cDNA Supermix Quanta kit (VWR, Mississauga, Canada). In preliminary experiments, three different sets of Iba-1 primers were tested; however, due to lack of specific amplification, we resorted to measure CD68, a marker of phagocytic microglia. For all PCR reactions, 500 nM (GFAP and CD68) and 300 nM (GUSB) of the forward and reverse primers were used. The PCR protocol and primer sequences are detailed in Additional file 1: Supplementary Methods.

### Immunofluorescence

Details on tissue preparation are available in Additional file 1: Supplementary Methods. Microglia were labeled with rat anti-mouse CD68 (1:10,000, AbDSerotec, Bio-Rad Laboratories, USA) or rabbit anti-Iba-1 (1:1000, Wako Inc., Richmond, USA) and astrocytes with mouse anti-GFAP primary antibodies (1:500, Invitrogen-Thermo Fisher Scientific, Burlington, Canada) as detailed in Additional file 1: Supplementary Methods. To minimize immunolabeling variations, sections from all groups in each batch were processed together. Immunolabeling specificity was assessed by omitting the primary antibodies. Confocal images were acquired with an Olympus laser-scanning microscope (model FV1000MPE). Two images per region per brain section, for a total of three sections per mouse, were captured for each condition. Expression levels of the markers of interest were analyzed in the hippocampal regions cornu ammonis 1 (CA1), cornu ammonis 3 (CA3), and dentate gyrus (DG) (from Bregma − 1.46 to − 1.70 mm), following previously published protocols [23]. Confocal images were imported into ImageJ (National Institutes of Health, Bethesda, USA), and the mean gray value tool was used to calculate relative fluorescence intensity units (RFU) in manually designed regions of interest (polygons of size 9.8E−5), computing an average RFU per section. Values were expressed relative to control group after background subtraction.

### Tissue processing for electron microscopy and ultrastructural analysis

Immunostaining was performed on sections containing the dorsal hippocampus (from Bregma − 1.46 to − 1.55 mm). Details on tissue processing and staining are available in the Additional file 1: Supplementary Methods. Pictures of Iba-1-stained microglial cell bodies and processes were randomly captured in the DG polymorphic layer at a magnification of × 6800 using an ORCA-HR bottom-mount digital camera (10 MP; Hamamatsu). For unbiased quantitative analysis, ~ 75 Iba-1-positive microglial processes per animal were analyzed by an observer blinded to the experimental conditions using ImageJ (National Institutes of Health, Bethesda, USA). The area, perimeter, and shape descriptor measurements circularity and solidity were used to assess changes in morphology. Vacuoles associated with autophagy and phagocytosis were also counted on a microglial process basis. In addition, the proportion of microglial processes associated with pockets of extracellular space containing cellular debris showing signs of digestion (i.e., shrinkage and increased width of the associated extracellular space), termed “extracellular digestion,” was determined.

### TNF-α and IL-6 ELISA

TNF-α and IL-6 were assessed by ELISA (mouse TNF-α kit #KMC3011 and mouse IL-6 kit #KMC0061, Invitrogen-Thermo Fisher Scientific, Burlington, Canada) in the brain or hippocampal homogenates as well as in plasma samples, as described in Additional file 1: Supplementary Methods. In order to combine data measured from separate kits, results were expressed as fold change with respect to the control group.

### Statistical analysis

Data analysis was done with GraphPad Prism v.7.0 (La Jolla, USA). Multiple group comparisons were evaluated by one-way or two-way analysis of variance (ANOVA), with the Tukey post hoc test. Two-group comparisons were analyzed with the Mann-Whitney test or a two-tailed *t* test, as appropriate. Spearman rank correlation was used to examine the association between TNF-α and blood pressure. A value of *p* ≤ 0.05 was considered statistically significant. For each experiment, the statistical analysis is specified in the figure legends and tables. Data is presented as mean ± SEM.

## Results

### Effect of Ang II doses on blood pressure

Subcutaneous perfusion of Ang II at a concentration of 1000 ng/kg/min resulted in a steady significant increase in systolic blood pressure (SBP) of 33.8 mmHg (mean increase) at day 4 and 37.1 mmHg (mean increase) at day 7, compared to day 0 (Table 1). In these conditions, a dose of Ang II of 200 ng/kg/min did not increase SBP even after 7 days of continued perfusion. The blood pressure of animals receiving Ang II 200 was also comparable to that of controls and will be therefore referred as subpressive. The blood pressure of animals receiving Ang II 1000 was greater than that of controls at days 4 and 7 and will be referred as pressive or hypertensive.

**Table 1:** Effect of Ang II doses on systolic blood pressure

	Control	Ang II 200	Ang II 1000
SBP day 0	132.0 ± 3.0	127.6 ± 3.5	125.3 ± 4.4
SBP day 4	132.1 ± 3.9	133.2 ± 4.3	159.1 ± 9.5^§¶^
SBP day 7	127.0 ± 4.1	129.0 ± 4.8	162.4 ± 8.3^§+^

Systolic blood pressure (SBP, mmHg) was assessed via the tail-cuff method. Mice were implanted with osmotic minipumps containing Ang II 200 ng/kg/min (referred as Ang II 200), Ang II 1000 ng/kg/min (referred as Ang II 1000), or 0.9% NaCl (referred as control). Data is expressed as mean ± SEM, *n* = 7–12/group; two-way ANOVA and Tukey post-test. Main effect of time *F*(2,92) = 5.203, *p* < 0.01; main effect of treatment *F*(2,92) = 12.95 *p* < 0.0001; interaction *F*(4,92) = 5.372, *p* < 0.001

^§^*p* < 0.001 Ang II 1000 day 4 and day 7 versus day 0, ^¶^*p* < 0.05 Ang II 1000 versus Ang II 200 and versus control, ^+^*p* < 0.001 Ang II 1000 versus Ang II 200 and versus control

### Effect of Ang II on cerebral gliosis

**Fig. 1:**
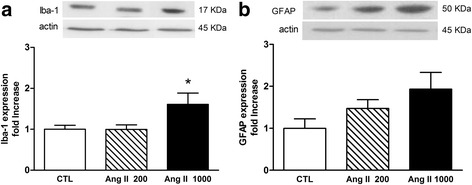
Effect of Ang II on cerebral gliosis. Iba-1 (microglia/monocyte marker) (**a**) and GFAP (astrocytic marker) (**b**) were examined by Western blotting in cerebral homogenates after 1 week systemic perfusion of Ang II 200 or 1000 ng/kg/min or 0.9% saline (CTL). Densitometry values of Iba-1 and GFAP were normalized to actin, and the results are expressed relative to the control group. Representative immunoblots are shown (*p < 0.05 Ang II 1000 versus 200 or controls, by one-way ANOVA with Tukey post-test, *n* = 9–15)

Mice receiving a hypertensive dose of Ang II exhibited greater levels of Iba-1 in the whole brain compared to controls (*p* = 0.035) or to mice receiving Ang II 200 (*p* = 0.034) (Fig. 1a, *n* = 9–12), indicating either an increase in the number of microglia or phenotypical changes of these cells. GFAP protein levels followed a similar trend in response to pressive Ang II (*p* = 0.076, Fig. 1b, *n* = 14–15). No significant changes in cerebral gliosis were detected in mice receiving subpressive doses of Ang II after 7 days perfusion (Fig. 1).

**Fig. 2:**
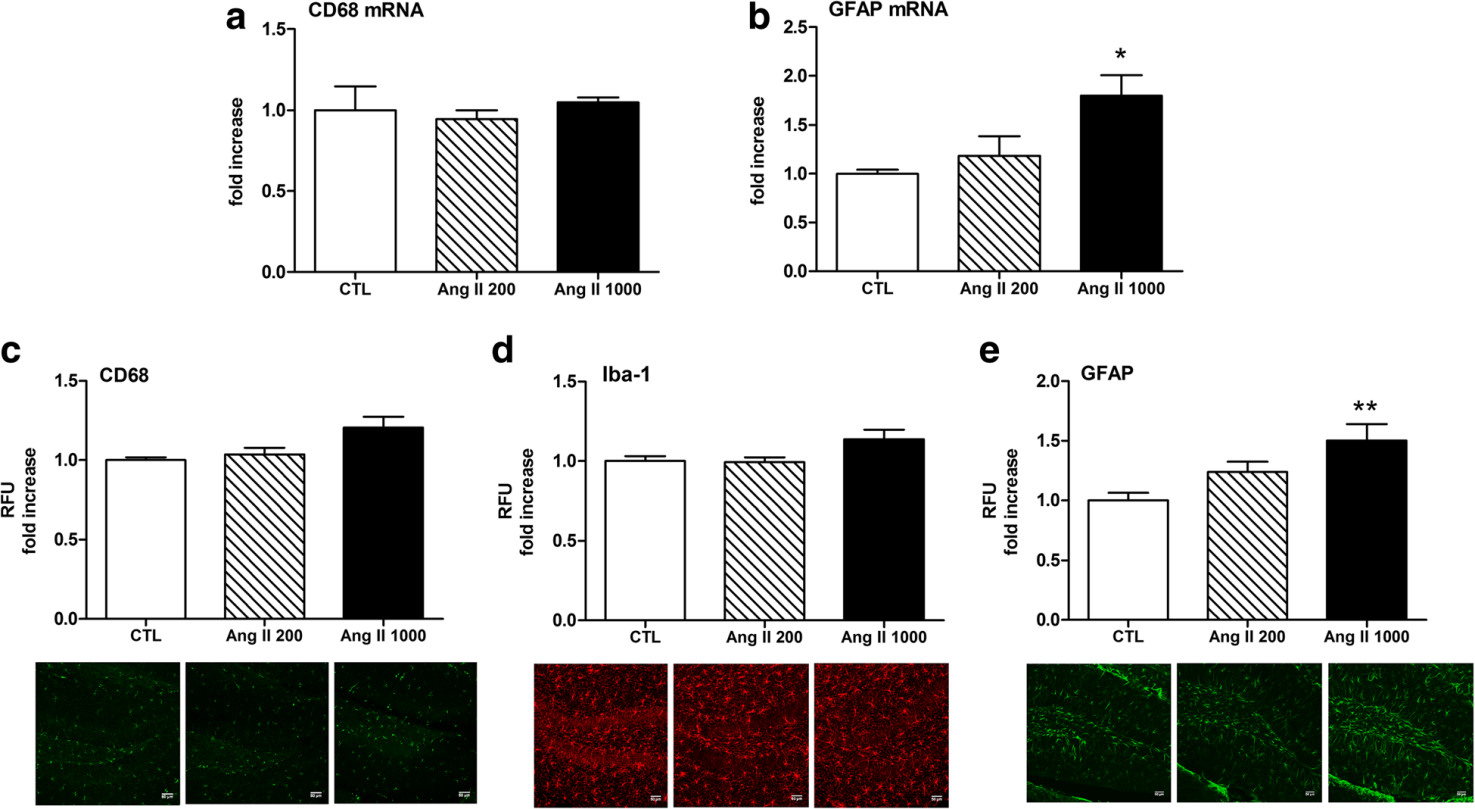
Effect of Ang II on hippocampal gliosis. CD68 mRNA (**a**) and GFAP mRNA (**b**) gene expression were analyzed by qRT-PCR in microdissected hippocampi after 1 week systemic perfusion of Ang II 200 or 1000 ng/kg/min or 0.9% saline (CTL). In each experiment, a treated-to-control ratio was calculated (*p < 0.05, Ang II 1000 versus control, by one-way ANOVA with Tukey post-test, *n* = 4–5). **c**–**e** Relative fluorescence intensity units (RFU) of CD68 (**c**), Iba-1 (**d**), and GFAP (**e**) in the hippocampus after 1 week systemic perfusion of Ang II 200 or 1000 ng/kg/min or 0.9% saline (CTL). In each experiment, a treated-to-control ratio was calculated (**p < 0.01, Ang II 1000 versus control, by one-way ANOVA with Tukey post-test, *n* = 4–6). Representative micrographs of CD68, Iba-1, and GFAP immunostainings in DG area of hippocampus are shown below each graph (scale 50 μm)

In the hippocampus, Ang II infusion did not increase CD68 mRNA synthesis regardless of the dose (Fig. 2a, *n* = 4–5). Instead, a significant increase in GFAP mRNA expression was evident in mice that received the hypertensive dose of Ang II (*p* = 0.031) but not the subpressive dose (Fig. 2b, *n* = 4–5). In line with the mRNA results, quantitative immunofluorescence showed comparable hippocampal CD68 levels between mice receiving subpressive Ang II and their controls, although a trend revealing a mean 16% increase in CD68 was seen with the pressive dose (*p* = 0.09, Fig. 2c, *n* = 4–6). Iba-1 showed a similar response (Fig. 2d, *n* = 4–6). We then examined the impact of Ang II on hippocampal astrogliosis. Immunofluorescence analysis revealed a significant increase in GFAP levels of around 50% in response to pressive Ang II (*p* = 0.001) but not to the subpressive dose (Fig. 2e).

To further distinguish the effects of Ang II and blood pressure on hippocampal gliosis, we used three different strategies. In the first one, we used a higher pressive Ang II dose (1900 ng/kg/min) that increased blood pressure to the same level of the dose of 1000 ng/kg/min. The second one examined the effects of phenylephrine, a hypertensive drug which is Ang II-independent. The third prevented Ang II-induced hypertension by simultaneously treating mice with hydralazine.

**Fig. 3:**
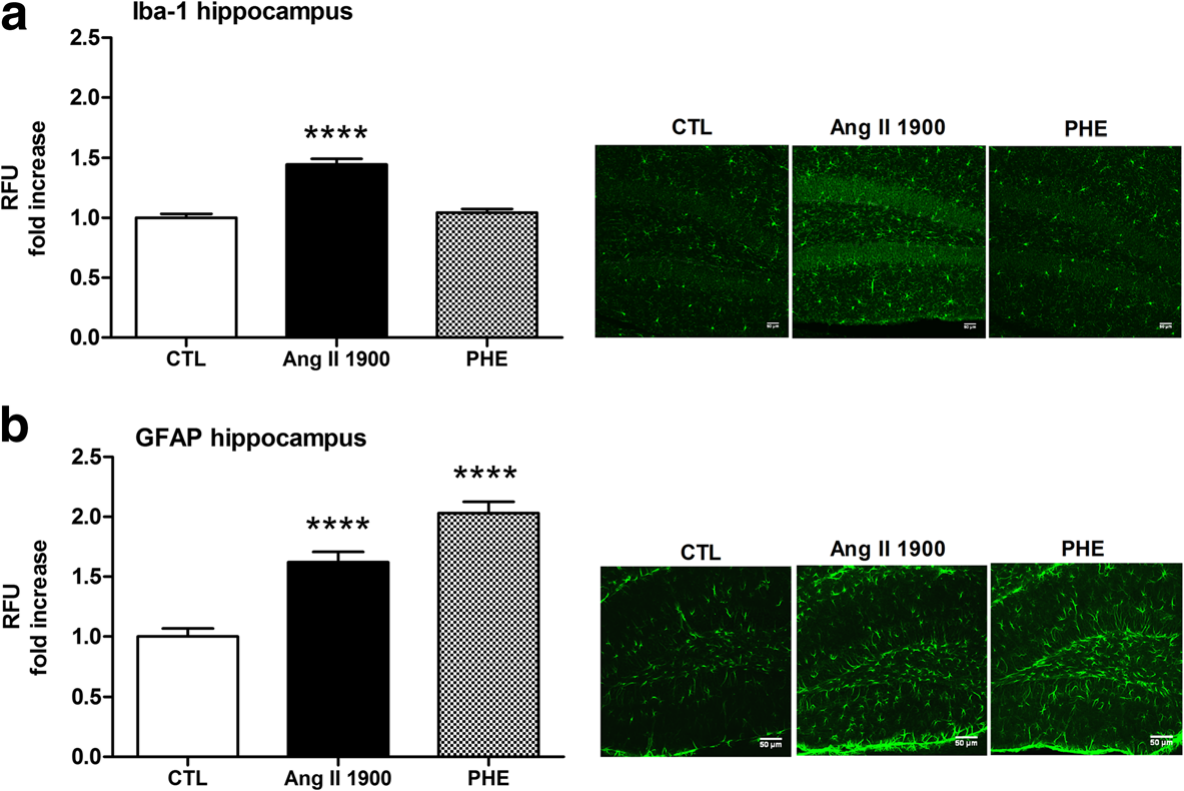
Effect of Ang II and phenylephrine on hippocampal gliosis. Iba-1 (**a**) and GFAP (**b**) were examined by immunofluorescence in the hippocampus after 1 week systemic perfusion of Ang II 1900 ng/kg/min or phenylephrine (PHE) 19 μg/kg/min or 0.9% saline (CTL). In each experiment, a treated-to-control ratio was calculated (****p < 0.0001, Ang II 1900 or PHE versus control, by one-way ANOVA with Tukey post-test, *n* = 4–5). Representative micrographs of Iba-1 and GFAP immunostainings in the DG area of the hippocampus are shown (scale 50 μm). RFU: relative fluorescence intensity units

Despite a similar increase of blood pressure as the dose of 1000 ng/kg/min (Tables 1 and 2), infusion of a higher pressive Ang II concentration (1900 ng/kg/min) led to a significant increase in hippocampal Iba-1 compared to controls (*p* < 0.0001, Fig. 3a, *n* = 4–5), suggesting a dose-dependent effect of Ang II on microgliosis. The higher dose of Ang II (1900 ng/kg/min) also increased GFAP levels by 60% following the 7-day chronic administration (*p* < 0.0001, Fig. 3b, *n* = 4–5).

**Table 2:** Effect of Ang II and phenylephrine on systolic blood pressure

	Control	Phenylephrine	Ang II 1900
SBP day 0	131.9 ± 3.2	132.3 ± 3.2	135.7 ± 1.0
SBP day 4	131.7 ± 2.2	142.5 ± 5.7^*^	158.5 ± 1.5^§¶^
SBP day 7	128.9 ± 0.5	145.6 ± 2.1^*#^	164.9 ± 2.3^§+^

Systolic blood pressure (SBP, mmHg) was assessed via the tail-cuff method. Mice were implanted with osmotic minipumps containing phenylephrine 19 μg/kg/min, Ang II 1900 ng/kg/min (referred as Ang II 1900), or 0.9% NaCl (referred as control). Data is expressed as mean ± SEM, *n* = 3/group; two-way ANOVA and Tukey post-test. Main effect of time *F*(2,12) = 15.64, *p* = 0.0005; main effect of treatment *F*(2,6) = 73.5 *p* < 0.0001; interaction *F*(4,12) = 7.252, *p* = 0.003.3

^*^p < 0.01 phenylephrine day 4 and day 7 versus control; ^#^*p* < 0.05 phenylephrine day 7 versus day 0; ^§^*p* < 0.001 Ang II 1900 day 4 and day 7 versus day 0; ^¶^*p* < 0.01 Ang II 1900 versus phenylephrine and versus control; ^+^*p* < 0.001 Ang II 1900 versus phenylephrine and versus control

No change in hippocampal Iba-1 expression was detected with perfusion of phenylephrine (Fig. 3a), even though it significantly raised blood pressure following the 7 days of chronic infusion (Table 2). Instead, phenylephrine significantly increased GFAP levels (*p* < 0.0001, Fig. 3b, *n* = 4–5), indicating that increased blood pressure alone can contribute to hippocampal astrogliosis.

**Fig. 4:**
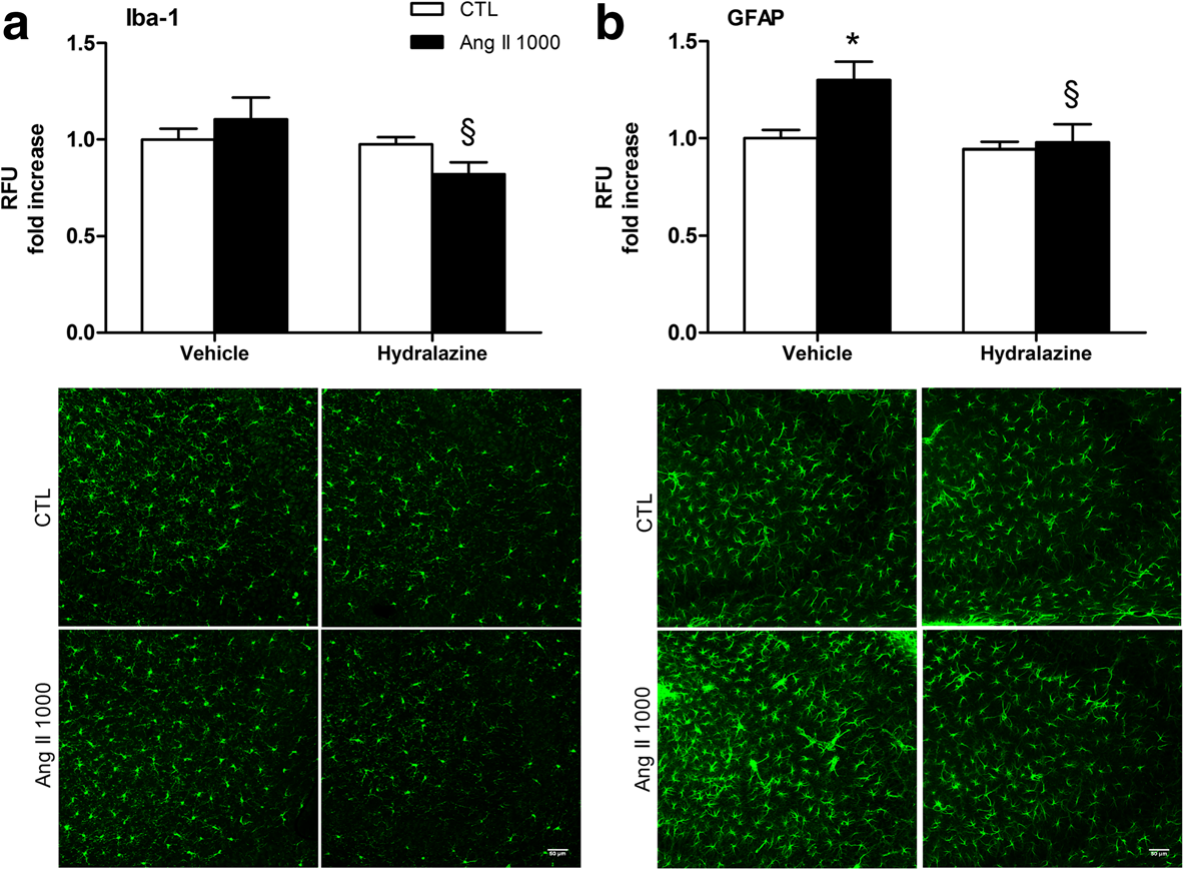
Effect of hydralazine on hippocampal gliosis induced by Ang II. Relative fluorescence intensity units (RFU) of Iba-1 (**a**) and GFAP (**b**) in the hippocampus after 1 week systemic perfusion of Ang II 1000 ng/kg/min or 0.9% saline (CTL). Hydralazine (150 mg/L) was administered in the drinking water starting 3 days before minipump implantation. Animals in the vehicle group received regular drinking water. In each experiment, a treated-to-control ratio was calculated (*p < 0.05 Ang II 1000 + vehicle versus control + vehicle; §*p* ≤ 0.05 Ang II 1000 + Hyd versus Ang II 1000 + vehicle, by two-way ANOVA with Tukey post-test, *n* = 3–8). Representative micrographs of Iba-1 and GFAP immunostaining in CA3 area of hippocampus are shown (scale 50 μm)

With a different strategy to explore the effects of blood pressure, we looked at Iba-1 and GFAP levels in the hippocampus of mice receiving the hypertensive dose of Ang II (1000 ng/kg/min) and treated with the peripheral vasodilator hydralazine (Fig. 4). Treatment with hydralazine effectively lowered blood pressure in hypertensive mice (Table 3). In line with earlier results quantitative immunofluorescence showed comparable hippocampal Iba-1 levels between mice receiving Ang II 1000 and their controls in this region (Fig. 4a). Treatment with hydralazine, which had no effect in control animals, resulted in a 25% reduction of Iba-1 in mice receiving Ang II, compared to those receiving the vehicle (*p* = 0.055, Fig. 4a, *n* = 3–6). Likewise, hydralazine reduced the elevation in hippocampal GFAP in mice receiving Ang II 1000 (*p* = 0.012, Fig. 4b, *n* = 6–8) to levels comparable to controls, supporting the idea that increased blood pressure is an important contributor to Ang II-induced astrogliosis.

**Table 3:** Effect of hydralazine treatment on hypertension induced by Ang II

	Control	Ang II	Control + Hyd	Ang II + Hyd
SBP day 0	127.0 ± 4.1	127.6 ± 2.5	119.4 ± 3.5	120.1 ± 3.6
SBP day 4	130.7 ± 3.7	165.4 ± 4.6^§^	118.9 ± 3.5	131.0 ± 4.0^¶^
SBP day 7	125.4 ± 3.8	171.4 ± 5.3^§^	115.2 ± 3.7	122.7 ± 2.6^¶^

Systolic blood pressure (SBP, mmHg) was assessed via the tail-cuff method. Mice were implanted with osmotic minipumps containing Ang II 1000 ng/kg/min or 0.9% NaCl (referred as control). Hydralazine (Hyd, 150 mg/L) was administered in the drinking water and started 3 days before minipump implantation. Data is expressed as mean ± SEM, *n* = 11–20/group; two-way ANOVA with Tukey post-test. Main effect of time *F*(2,191) = 11.58, *p* < 0.0001; main effect of treatment *F*(3, 191) = 49.42, *p* < 0.0001; interaction *F*(6, 191) = 8.772, *p* < 0.0001

^§^*p* < 0.0001 Ang II day 4 and day 7 versus day 0 and versus control; ^¶^*p* < 0.0001 Ang II + Hyd versus Ang II on day 4 and day 7

To rule out that the absence of brain inflammatory changes in mice receiving the subpressive dose for 1 week was not due to insufficient perfusion time, we examined a separate group of mice that received chronic Ang II infusion (200 ng/kg/min) during 14 days. Western blot analysis in the whole brain revealed a trend for a mean 35% increase in Iba-1 levels (*p* = 0.065, Additional file 2: Figure S1A, *n* = 3) and no differences in GFAP (Additional file 2: Figure S1B, *n* = 3). While no differences in hippocampal CD68 had been seen with infusion of subpressive Ang II for 1 week, increasing Ang II perfusion to 2 weeks led to a significant rise in CD68 mRNA (mean increase 27%, *p* = 0.0090, Additional file 2: Figure S1C, *n* = 7) and to a similar increase in GFAP mRNA (mean increase 29%), although this difference did not reach statistical significance (*p* = 0.117, Additional file 2: Figure S1D, *n* = 7).

### Effect of hypertensive Ang II on microglial morphology

**Fig. 5:**
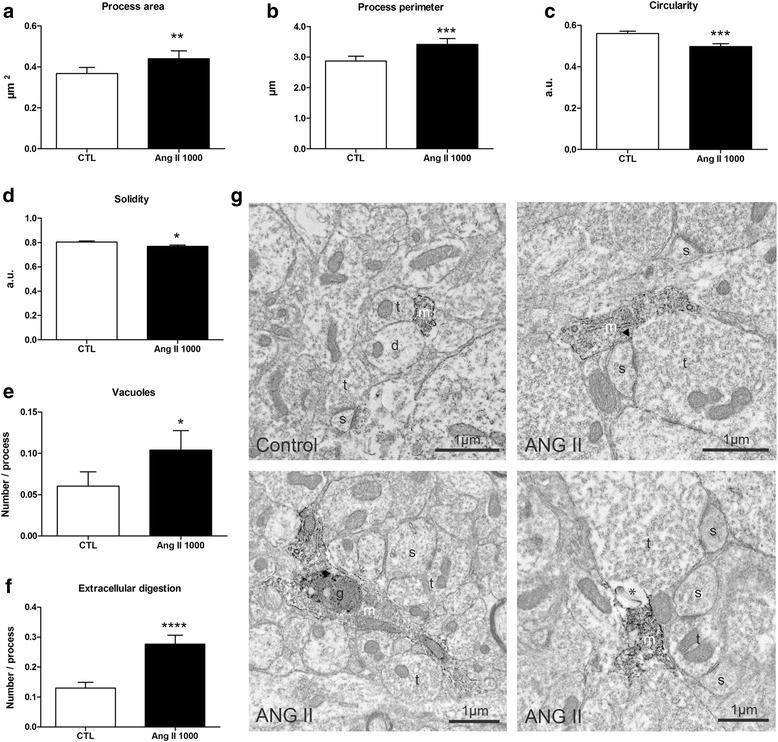
Effect of Ang II on microglial process ultrastructure. Differences in microglial process ultrastructure between control (CTL) and Ang II 1000 ng/kg/min treated animals, as observed within the hippocampal DG polymorphic layer. The area (**A**), perimeter (**B**), circularity (**C**), and solidity (**D**) were used to assess changes in morphology. Vacuoles (**E**) indicative of autophagy or phagocytosis were counted on a microglial process basis. The proportion of microglial processes associated with pockets of extracellular space containing cellular debris showing signs of digestion, termed “extracellular digestion” (**F**) was also determined (*p < 0.05, **p < 0.01, ***p < 0.001, ****p < 0.0001, by Mann-Whitney test, *n* = 3–4) a.u.= arbitrary units. **G** Representative pictures of Iba-1-stained microglial cell bodies and processes captured at a magnification of × 6800. Typical Iba-1-positive microglial process (m) observed in a control animal (upper left). Examples of Iba-1-positive microglial processes in Ang II-treated animals (upper right and lower panels). The microglial process is making direct contacts with a pre-synaptic axon terminal (t), a synaptic cleft (arrowhead), and a post-synaptic dendritic spine (s) (upper right). The elongated microglial process with a complex morphology that contains an accumulation of lipofuscin granules, a hallmark of cellular aging (g) (lower left). The microglial process is associated with a pocket of extracellular space that contains cellular debris undergoing digestion (asterisk). d dendrite (lower right)

To better characterize the impact of hypertensive Ang II on cerebral gliosis, we performed a detailed quantitative analysis of microglial morphology in the hippocampus using immuno electron microscopy (EM). We studied ultrastructural changes in microglial processes (area, perimeter, circularity, and solidity) as well as changes in their accumulation of vacuoles and extracellular digestion. EM analysis showed significant differences in microglia morphology between control and hypertensive mice receiving Ang II 1000 ng/kg/min (*n* = 3–4). There was a 19% mean increase in both process area (*p* = 0.002, Fig. 5a) and perimeter (*p* = 0.0002, Fig. 5b), indicating that processes are larger (“thickened”) in the presence of Ang II. We also observed significant reductions in circularity (*p* = 0.0005, Fig. 5c), indicating that processes have more complex shapes in the presence of pressive Ang II. Solidity, a measure of the indentations on the membrane of processes, was also reduced in animals infused with hypertensive Ang II (*p* = 0.011, Fig. 5d), indicating that microglia are more ruffled. This could be an indicator of increased motility. Electron microscopy also revealed significant increases in the abundance of microglial vacuoles (mean increase 72%, *p* = 0.046, Fig. 5e), indicating the occurrence of autophagy or phagocytic degradation and increased extracellular digestion in Ang II-infused mice (mean increase 112%, *p* < 0.0001, Fig. 5f).

### Effect of Ang II on cerebral pro-inflammatory cytokine production

**Fig. 6:**
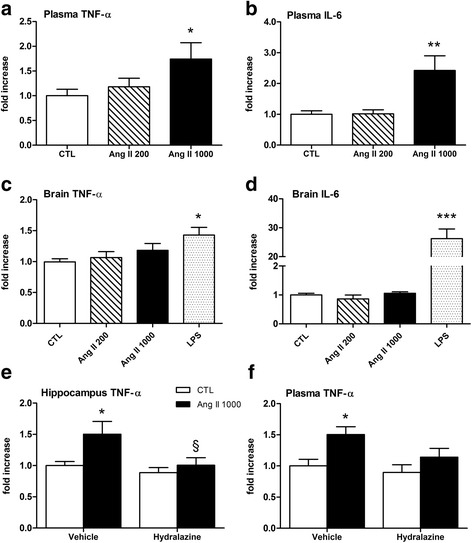
Effect of Ang II on cerebral and systemic cytokine production. ELISA analysis of TNF-α and IL-6 in plasma (**a**, **b**) and whole brain homogenates (**c**, **d**) after 1 week perfusion of Ang II 200, 1000 ng/kg/min or 0.9% saline (CTL). LPS (2 mg/kg) was injected i.p. and mice were sacrificed 3 h after. A treated-to-control ratio was calculated. (*p < 0.05, **p < 0.01, ***p < 0.001 versus control, by one-way ANOVA with Tukey post-test, *n* = 6–8). **e**, **f** ELISA analysis in hippocampus (**e**) and plasma (**f**) of mice receiving Ang II 1000 ng/kg/min or 0.9% saline (CTL) for 1 week. Hydralazine (150 mg/L) was administered in the drinking water and started 3 days before minipump implantation. Animals in the vehicle group received regular drinking water. Results are expressed a treated-to-control ratio. (*p < 0.05 Ang II + vehicle versus control + vehicle, §*p* < 0.05 Ang II + Hyd versus Ang II + vehicle by two-way ANOVA with Tukey post-test, *n* = 6–9)

We next examined whether systemic Ang II would lead to increased production of pro-inflammatory cytokines in the brain. First, we confirmed that Ang II infusion increased the levels of the pro-inflammatory mediators TNF-α and IL-6 in plasma. As shown in Fig. 6a, b, hypertensive doses of Ang II led to significant increases in the circulating levels of TNF-α (*p* = 0.022 versus control and *p* = 0.067 versus Ang II 200, *n* = 6) and IL-6 (*p* = 0.004 versus control and *p* = 0.003 versus Ang II 200, *n* = 6–8). Mice that received Ang II 200 ng/kg/min did not present elevations in peripheral plasma cytokines, neither after 1 week nor after 2 weeks perfusion (Fig. 6a, b and Additional file 2: Figure S1E, *n* = 6–8).

Despite the robust systemic pro-inflammatory effect seen with hypertensive Ang II, ELISA analysis did not detect an elevation of these cytokines in the whole brain with any of the doses (Fig. 6c, d, *n* = 6–8). This was not due to a lack of detection capacity of our method as mice receiving a single systemic LPS injection showed significant increases in the production of TNF-α (1.4-fold, *p* = 0.047) and of IL-6 (26-fold, *p* < 0.0001) compared to controls (Fig. 6c, d, *n* = 7). Even with longer perfusion times, no changes in TNF-α production in the whole brain were detected with infusion of Ang II 200 ng/kg/min for 2 weeks (Additional file 2: Figure S1F, *n* = 6–8).

### Effect of Ang II on TNF-α production in the hippocampus

We next considered whether the apparent absence of increased cytokine production in the brain of mice chronically infused with Ang II was due to the presence of a specific regional change, which was diluted by analysis of the whole brain. We decided to examine TNF-α in more detail rather than IL-6, based on previous evidence that hypothalamic microglia isolated from Ang II-treated mice exhibit a significant increase in TNF-α expression and a milder increase in IL-6 [17, 18]. In line with the proposed hypothesis, ELISA analysis of hippocampal homogenates revealed a significant increase in TNF-α levels in mice infused with Ang II 1000 ng/kg/min compared to controls (*p* = 0.040, Fig. 6e, *n* = 6–9). Supporting the effects of blood pressure on cerebral inflammation, hydralazine treatment prevented the increase in hippocampal TNF-α production triggered by hypertensive Ang II (*p* = 0.037, Fig. 6e), with a similar trend in the periphery (Fig. 6f, *n* = 6–9).

We further examined the association between hippocampal TNF-α and systolic blood pressure via correlation analysis. While a significant positive association was found between the concentration of TNF-α measured in plasma and blood pressure (*r* 0.658, *p* = 0.017), Spearman analysis did not reveal a significant correlation between these two parameters when TNF-α was assessed in the brain (*r* 0.456 *p* = 0.102), suggesting a non-linear relationship between blood pressure and cerebral inflammation.

## Discussion

This study presents several novel findings. First, it provides new knowledge on specific brain regions involved in Ang II-induced inflammation beyond the PVN, by showing increased TNF-α production, elevated GFAP expression, and microgliosis in the hippocampus. Second, it presents a thorough quantitative characterization of hippocampal microglia morphology achieved via EM, providing a better understanding of their functional activation state in relation to hypertension. Third, it reveals that both increased blood pressure and Ang II concentrations are important contributors to hippocampal inflammation induced by systemic Ang II.

Ang II, the main effector of the renin-angiotensin system, is a circulating peptide hormone and a potent vasoconstrictor that exerts key roles in the regulation of blood pressure and the development of hypertension [24]. The brain also has its own intrinsic renin-angiotensin system from where Ang II is produced [25–28]. In rodents and humans, Ang II binding sites are expressed widely throughout the brain including the cerebral vasculature and circumventricular organs (CVO), where they have access to systemic Ang II [27, 29, 30]. Within the brain parenchyma, angiotensin II type I (AT1) receptors are highly expressed in neurons among specific brain regions involved in autonomic and cardiovascular regulation and, although in lower levels, in other areas including the cerebral cortex, hippocampus, basal ganglia, and cerebellum [27, 29, 30].

Whether systemic Ang II and increased blood pressure contributed to cerebral inflammation beyond the cerebral vasculature and PVN was not clear before this study. Therefore, we delivered pressive and subpressive doses of Ang II and used phenylephrine or hydralazine, which modulate blood pressure independently of the renin angiotensin system (phenylephrine increases blood pressure via α-1 adrenergic receptors, and hydralazine lowers blood pressure by relaxing vascular smooth muscle). With these approaches, our results show that circulating Ang II leads to hippocampal inflammation and cerebral gliosis in a dose-dependent manner and that increased blood pressure is an important contributor to the brain pro-inflammatory effects of Ang II in mice.

Is blood pressure elevation alone sufficient? With administration of phenylephrine, we did not observe an increase in Iba-1, as we have seen with the highest hypertensive dose of Ang II, even though the dose of phenylephrine was ten times higher than that of Ang II (19 μg/kg/min versus 1900 ng/kg/min). In addition, analysis showed a non-linear correlation between hippocampal TNF-α and blood pressure, suggesting that other factors could modulate the association between these parameters. For example, the breakdown of the blood-brain barrier (BBB) induced by Ang II may be involved in the inflammatory process [31, 32]. Indeed, a study from Marvar showed that Ang II is critical for BBB damage, independently of blood pressure [31]. The effects of Ang II are also highlighted in this study by the fact that hippocampal Iba-1 levels rose with increasing concentrations of Ang II, while the increase in blood pressure produced by these hypertensive doses was similar. Likewise, increasing subpressive Ang II (200 ng/kg/min) infusion duration to 14 days resulted in a significant increase in hippocampal CD68 mRNA, with a similar trend for Iba-1, without changes of GFAP or TNF-α. These results emphasize the importance of Ang II actions and also suggest that a certain threshold of blood pressure may be required for the full expression of cerebral inflammation.

Despite bypassing the renin-angiotensin system, we observed a significant increase in GFAP expression in the hippocampus with phenylephrine treatment. This vulnerability of astrocytes is not surprising considering their anatomical proximity to cerebral blood vessels as part of the neurovascular unit [33], being close targets of the pressor stress suffered by the vasculature in hypertension. Indeed, it is known that astrocytes can act as “sensors” to vascular damage, by expressing ion channels sensitive to shear stress, such as TRPV4 (transient receptor potential vanilloid subtype 4) [34], which when activated lead to the development of astrogliosis [35].

Beyond our study, a critical role for Ang II on cerebral inflammation is supported by a previous work showing that inhibition of AT1 receptors with candesartan, a centrally acting angiotensin receptor blocker, leads to reductions in brain inflammation induced by LPS or stroke in normotensive rats [36, 37]. At the same time, our results on blood pressure complement the findings of Marvar and colleagues who used a similar approach with hydralazine to demonstrate that the pressor effects of Ang II are critical for the activation of circulating T lymphocytes and the expression of vascular inflammation in mice [31]. Interestingly, there is also evidence that hydralazine attenuates cardiac fibrosis and inflammation induced by pressive Ang II in mice, suggesting that blood pressure is also a key contributor to the expression of inflammation in other end organs affected by hypertension besides the brain [38].

With respect to hydralazine, there have been reports indicating it exhibits antioxidant properties beyond its well-recognized antihypertensive effect, including scavenger actions on peroxynitrite and oxygen radicals [39–41]. Although it can be argued that attenuation of oxidative stress by this peripheral vasodilator could have helped diminish the cerebral inflammation caused by hypertensive Ang II, some considerations reaffirm its main mechanism of action through blood pressure reduction. First, the range of hydralazine doses with antioxidant properties previously reported was much higher than the dose used in this study (≥ 250 versus 150 mg/L, respectively). Second, although low hydralazine doses (similar or lower than this study) demonstrated ROS scavenging actions in vitro (reductions of oxidative radicals by 25%), this effect was significantly lower compared to classical antioxidants such as ascorbic acid, which reduced oxygen radical production by 80% [39]. Third, in the discussed studies, the antioxidant actions of hydralazine have been evaluated in vitro in peritoneal macrophages, cultured smooth muscle cells, and isolated vessels, or ex vivo in isolated rabbit aortas. Therefore, to the best of our knowledge, the cerebral antioxidant actions of hydralazine remain to be demonstrated.

Previous studies examining the cerebral pro-inflammatory effects of pressive Ang II focused on the PVN, a region of the hypothalamus involved in the sympathetic control of blood pressure [15]. These studies showed changes in microglial morphology (enlarged soma, process retraction) and increased production of pro-inflammatory cytokines (IL-1β, IL-6, and TNF-α) [16–18]. Our results confirm that pressive Ang II leads to cerebral gliosis and extends this finding to another brain region, namely the hippocampus. Our work also complements the study of Toth and colleagues who demonstrated that the hippocampal expression of the chemokines MCP-1 (CCL2) and IP-10 (CXCL10) is increased in young mice (3 months) receiving pressive doses of Ang II (1000 ng/kg/min, 28 days), a response that is exacerbated by aging (24 months), where upregulation of CD68 and TNF-α then became evident [42].

Besides an upregulation of TNF-α and astrogliosis, we report significant morphological changes of microglia in the hippocampus of mice infused with pressive Ang II, as detected by immuno-EM. Changes in microglial morphology are informative of their functional activation state in response to stimuli or injury. In this study, we labeled microglia with Iba-1, which is a well-recognized marker specifically expressed by resident microglia and macrophages [43, 44]. We report significant changes in quantitative shape and functional descriptors including increases in microglial process area and perimeter, reductions in circularity and solidity, and a greater number of vacuoles and increased extracellular digestion.

The increase in process area and perimeter would indicate that they are larger in the presence of Ang II, reflecting process thickening and therefore increased metabolic or lysosomal activity. This is also supported by the finding of reduced solidity and thus of a more ruffled morphology, which could be a sign of increased process motility reflecting their capacity to reorient towards sites of injury [45]. Furthermore, the changes in vacuoles and increased extracellular digestion suggest an involvement of microglia in synaptic remodeling in the presence of Ang II. This is supported by the EM micrographs showing microglial processes with complex morphologies making contacts with pre-synaptic axon terminals, synaptic clefts, and post-synaptic dendritic spines and containing cellular debris being degraded. A seminal study recently showed that the physiological functions of microglia that prune excess synapses during development are aberrantly activated in Alzheimer’s disease animal models, contributing to synaptic loss, which is a strong correlate of cognitive decline [46]. It is therefore possible that microglia in the presence of Ang II undergo phenotypic changes that lead to inflammation, synaptic pruning, and ultimately cognitive impairments. Indeed, these specific changes in the hippocampus are in line with our earlier findings that pressive Ang II leads to cognitive dysfunctions in tasks that are dependent on this region [22]. They are also consistent with a recent study examining the impact of Ang II (1000 ng/kg/min, 28 days perfusion) on synaptic plasticity in mice, showing that systemic Ang II leads to reductions in hippocampal long-term potentiation, decreased density of hippocampal synapses, and reduced expression of key genes involved in synaptic plasticity, such as *Bdnf* and *Homer1* [47]. We could thus speculate that the development of hippocampal inflammation and synaptic dysfunction is a mechanistic link between early hypertension and the brain deficits that lead to neuronal injury and dementia in late-life [19–21].

It is interesting that such significant and specific changes in hippocampal microglia morphology were detected in the absence of increased expression of Iba-1 or CD68 in this region. These results highlight the sensitivity of immuno-EM ultrastructural studies to reveal alterations in microglia morphological dynamics that may otherwise appear “normal” when examined by protein or gene expression analysis. With respect to other regions, the increase in Iba-1 in the whole brain in response to pressive Ang II could be a signal of increased microglial numbers or phenotypic alterations taking place outside the hippocampus (e.g., the PVN, as previously shown) or even in the white matter, an interesting hypothesis warranting future investigations.

In addition to maintaining brain homeostasis, microglia undergo phenotypic transformation upon tissue injury, or in response to Ang II, leading to the secretion of inflammatory and oxidative molecules that contribute to neurodegeneration [48]. A recent study demonstrated that isolated hypothalamic microglia actually respond to Ang II through AT1 and TLR4 receptors, the latter which is required for the expression of oxidative damage induced by Ang II in mice [49]. Although the role of other cells including neurons and astrocytes cannot be discarded, the ultrastructural morphological changes along with the increased hippocampal TNF-α production suggest the emergence of a pro-inflammatory microglial phenotype in mice with chronic Ang II administration. This is supported by Shen and colleagues who showed that the increase in hypothalamic TNF-α that results from in vivo Ang II administration returned to normal levels when microglia were depleted from the brain [17].

Besides affecting microglia, our finding of increased hippocampal GFAP expression in response to pressive Ang II indicates that astrocyte activation is also involved in Ang II-induced neuroinflammation. Indeed, in a very comprehensive study, Stern and colleagues recently showed that astrocytes are critical and direct cellular mediators of Ang II actions on PVN neurons, leading to increased sympathetic outflow and to increased blood pressure [50].

Therefore, it is likely that the cerebral inflammation induced by Ang II involves multiple pathways and cellular players, including but not limited to (i) the direct effects of circulating Ang II on AT1 receptors in cerebral vessels [51]; (ii) the damage to the BBB [32], which could facilitate the entry of Ang II to brain regions beyond the CVO network (i.e., the hippocampus); (iii) the consequent effects on microglia and astrocytes, leading to the expression of pro-inflammatory cytokines within the brain; and (iv) the entry of activated immune cells from the periphery.

The findings of our study must be considered within its limitations. One lesson learned from our work is that microglia morphology analysis via EM is able to detect subtler and even more informative changes than immunofluorescence, as seen by the lack of Iba-1 increase with the dose of 1000 ng/kg/min Ang II in parallel with significant changes in the morphology of microglia. In this regard, although Iba-1 levels in phenylephrine-treated animals were comparable to controls, we cannot discard the possibility that phenylephrine could have affected microglia morphology in this group. It would be interesting to examine whether metabolic changes in microglia differ upon Ang II or phenylephrine treatment despite similar effects on blood pressure. Also, our study examined only male mice and potential sex differences in the response to the pressive and inflammatory effects of Ang II cannot be discarded. Despite these considerations, this work broadens our understanding of the in vivo effects of Ang II actions in relation to hypertension and its impact on brain inflammation.

## Conclusions

Our results indicate that the cerebral pro-inflammatory effects of pressive Ang II involve changes in microglial morphology, as well as increases in cytokine production and GFAP expression in a region of the brain critical for memory. EM analysis revealed phenotypic alterations in microglia consistent with an active metabolic state, which could be involved in synaptic remodeling, in the absence of increases in Iba-1 or CD68 expression. This emphasizes the value of immuno-EM to reveal alterations in cerebral gliosis upon tissue injury. The findings of this study also indicate that increased blood pressure, in addition to Ang II itself, is an important contributor to the development of cerebral inflammation. Taken together, these results open the door for future investigations on the mechanisms underlying the induction of cerebral inflammation by hypertension, leading to new questions such as the following: What is the threshold of blood pressure necessary to induce cerebral inflammation? Which parameters influence this threshold? Can antihypertensive treatments reverse this inflammation? Answers to these questions may provide great advancements for a better understanding of the link between hypertension and cognitive decline as well as for the development of therapies against neurodegenerative diseases.

## Additional files

Additional file 1: Supplementary Methods. (DOCX 59 kb)

Additional file 2: **Figure S1.**. Effect of extended perfusion of subpressive Ang II on cerebral inflammation. Iba-1 (A) and GFAP (B) were examined by Western blotting in cerebral homogenates after 2 weeks systemic perfusion of Ang II 200 ng/kg/min or 0.9% saline (CTL). Densitometry values of Iba-1 and GFAP were normalized to actin, and the results are expressed relative to the control group. Representative immunoblots are shown (two-tailed Student’s *t* test, *n* = 3). CD68 mRNA (C) and GFAP mRNA (D) gene expression were analyzed by qRT-PCR in microdissected hippocampi after 2 weeks systemic perfusion of Ang II 200 ng/kg/min or 0.9% saline (CTL). In each experiment, a treated-to-control ratio was calculated (**p < 0.01, Ang II 200 versus control, by two-tailed Student’s *t* test, *n* = 7). ELISA analysis of TNF-α in plasma (E) and in whole brain homogenates (F) after 2 weeks perfusion of Ang II 200 ng/kg/min or 0.9% saline (CTL). A treated-to-control ratio was calculated (two-tailed Student’s *t* test, *n* = 6–8). (TIFF 1442 kb)

## Abbreviations

Ang II: Angiotensin II
AT1: Angiotensin II type I
a.u: arbitrary units
BBB: Blood-brain barrier
CA1: Cornu ammonis 1
CA3: Cornu ammonis 3
CVO: Circumventricular organs
DG: Dentate gyrus
DT: Diphtheria toxin
EM: Electron microscopy
GFAP: Glial fibrillary acidic protein
Hyd: Hydralazine
Iba-1: Ionized calcium-binding adaptor molecule 1
IL-1β: Interleukin-1β
IL-6: Interleukin 6
LPS: Lipopolysaccharides
PVN: Paraventricular nucleus
RFU: relative fluorescence intensity units
SBP: Systolic blood pressure
TNF-α: Tumor necrosis factor-α
