# Supplementary material for: Differential effect of angiotensin II and blood pressure on hippocampal inflammation in mice
Source: J Neuroinflammation. 2018 Feb 28;15:62. doi: 10.1186/s12974-018-1090-z (PMC6389185; doi:10.1186/s12974-018-1090-z)
Supplement: Supplementary file 1 — Supplementary Methods. (DOCX 59 kb) [file 12974_2018_1090_MOESM1_ESM.docx]

**Additional file methods**

**Western blotting**

Following perfusion with cold phosphate-buffered saline (PBS), the brains were isolated, frozen and homogenized in RIPA lysis buffer (50 mM Hepes, 150 mM NaCl, 5 mM EGTA, 2 mM MgCl_2_, 5% glycerol, 1% Triton X-100, 0.1% SDS, 1% Na-deoxycholate, pH 7.4) containing protease and phosphatase inhibitors (Roche, Indianapolis, USA). Equal amounts of proteins (75 µg) were loaded on SDS-polyacrylamide gels that were subjected to electrophoresis and then transferred to nitrocellulose membranes (BioRad Laboratories, ON, Canada). Membranes were probed with rabbit anti-Iba-1 (1:500, Wako Inc, Richmond, USA) or mouse anti-GFAP (1:2000, (EMD Millipore, Maine, USA) primary antibodies overnight at 4°C. Rabbit anti-pan actin (1:1000, New England Biolabs, Ltd, Whitby, Canada) was used as loading control. Secondary antibodies (goat anti-rabbit 1:1000 or rabbit anti-mouse 1:5000, EMD Millipore, Maine, USA) were applied for 1 hour. The immunoreactive proteins were detected with an enhanced chemiluminescence substrate (ECL, Invitrogen-Thermo Fisher Scientific, Burlington, Canada) and developed on autoradiography films. Densitometric analysis was performed using ImageJ software (National Institutes of Health, Bethesda, USA) and the ratio Iba-1/actin or GFAP/actin was expressed relative to the control group.

**RNA extraction and quantitative real time PCR (qRT-PCR)**

Mice were anesthetized with sodium pentobarbital (100 mg/kg, CDMV, Saint-Hyacinthe, Canada) and decapitated. The hippocampus was carefully dissected. Total RNA was extracted from mouse hippocampi using the RNeasy Plus Universal mini kit (Qiagen, Toronto, Canada) in accordance with instructions of the manufacturer. cDNA was synthesized with the qScript cDNA Supermix Quanta (VWR, Mississauga, Canada). For all PCR reactions, 500 nM (GFAP and CD68) and 300 nM (GUSB) of the forward and reverse primers were used. The RT-PCR reaction was performed using the SYBRGreen dye in an Illumina Eco Instrument (Illumina Inc., USA). PCR was performed by initial denaturation at 95 °C for 5 min, followed by 40 cycles of 15 sec at 95 °C, 30 sec at 62 °C, and 30 sec at 72 °C. Relative differences in gene expression were normalized to that of the housekeeping gene GUSB (beta-glucuronidase). Threshold cycle (Ct) values were used for data analysis. The sequences of the primers used were as follows: For CD68: Fwd GCTACATGGCGGTGGAGTACAA and Rev ATGATGAGAGGCAGCAAGATCG, for GFAP: Fwd ACATCGAGATCGCCACCTAC and Rev TGCTTCGACTCCTTAATGAC for GUSB: Fwd CAGGGTCAACTTCAGGTTCC and Rev GCTCTTTGTGACAGCCACTG.

**Immunofluorescence**

Mice were anesthetized with sodium pentobarbital (100 mg/kg, CDMV, Saint-Hyacinthe, Canada) and perfused transcardially with 10 mL of PBS (pH 7.4) followed by 50 mL 4% paraformaldehyde (PFA, Bioshop, Burlington, Canada). The brains were removed, post-fixed in 4% PFA at 4°C for 24 hours and cut into 40 µm-thick coronal sections with a vibratome (Leica, VT1000s). The sections were kept in an antifreeze solution containing 30% glycerol, 30% ethylene glycol in PBS at -20°C. Brain sections were placed in a PBS blocking solution for 1 hour at room temperature containing 10% bovine serum albumin (BSA), 5% normal goat serum (NGS) and 0.3% Triton X-100. Microglia were labeled with rat anti-mouse CD68 (1:10000, AbDSerotec, Bio-Rad Laboratories, USA) and rabbit anti-Iba-1 antibody (1:1000, Wako Inc, Richmond, USA) and astrocytes with GFAP primary antibody coupled to Alexa Fluor 488 (1:500, Invitrogen-Thermo Fisher Scientific, Burlington, Canada) for 24 hours at 4°C following previously described protocols [1]. Secondary antibodies Alexa fluor 488 goat anti-rat (1:500) for CD68 and Alexa fluor 488 goat anti-rabbit or Alexa fluor 633 goat anti-rabbit for Iba-1 (1:500, Invitrogen-Thermo Fisher Scientific, Burlington, Canada) was applied for 2 hours at room temperature. For GFAP labeling, antigen retrieval was done by heating brain sections in a microwave oven 15 times for 30 seconds at 30% power with 5 min intervals, in a 10 mM citrate solution pH 6.0. Then sections were incubated with GFAP primary antibody coupled to Alexa Fluor 488 (1:500, Invitrogen-Thermo Fisher Scientific, Burlington, Canada) for 48 hours at 4°C. To assure uniformity of the stainings, sections from all groups in each batch were processed together. The specificity of all immunolabels was assessed by omitting the primary antibodies. Confocal images were acquired with an Olympus laser-scanning microscope (model FV1000MPE) using the same acquisition parameters and appropriate filters. Two images per region per brain section, for a total of three sections per mouse, were captured for each condition. Expression levels of the markers of interest were analyzed in the hippocampal regions CA1, CA3 and DG (from Bregma -1.46 to -1.70 mm). Confocal images were imported into ImageJ (National Institutes of Health, Bethesda, USA) and the mean gray value tool was used to calculate relative fluorescence intensity units (RFU) in manually designed regions of interest (polygons of size 9.8E-5), computing an average RFU per section Values were expressed relative to control group after background subtraction.

**Tissue processing for electron microscopy and ultrastructural analysis**

Mice were anesthetized with sodium pentobarbital (100 mg/kg) and perfused transcardially with 15 mL of cold PBS (pH 7.4) followed with 75 mL 3.5% acrolein (Sigma-Aldrich, Oakville, Canada) and then 150 ml 4% PFA as previously described [2]. The brains were removed, post-fixed for at least 2 hours in 4% PFA at 4°C and then cut into 50 μm-thick coronal sections using a vibratome (Leica VT 1000S). Sections were stored in antifreeze solution (30% glycerol, 30% ethylene glycol, in PBS 0.05M, pH 7.4) at -20°C until processing. Immunostaining was performed on sections containing the dorsal hippocampus (from Bregma -1.46 to -1.55 mm), selected using Paxinos and Franklin's mouse brain atlas.

Sections were incubated with 0.3% H_2_O_2_ in PBS for 5 min and then transferred to 0.1% NaBH_4_ in PBS for 30 min. Afterwards, they were incubated in a blocking solution (10% FBS, 3% BSA, 0.01% Triton X-100) for 1 hour followed by overnight incubation with rabbit anti-Iba-1 primary antibody (1:1000; Wako Inc, Richmond, USA) diluted in blocking solution at 4°C. The next day, sections were washed in PBS and kept in goat anti-rabbit antibody conjugated to biotin (Jackson Immunoresearch, West Grove, USA) diluted 1:300 in TBS (0.05M, pH 8.0) for 90 min. They were then processed with the Vectastain ABC kit (Vector Laboratories, Burlingame, USA). The staining was revealed with a solution of 0.05% diaminobenzidine (DAB) and 0.015% H_2_O_2_ diluted in TBS (0.05M, pH 8.0). After, sections were treated with osmium tetroxide (1% in PB 0.1M, pH 7.4) and later dehydrated in increasing concentration of ethanol (35%, 50%, 70%, 90%, 100%), followed by propylene oxide to remove residual water before embedding in Durcupan resin overnight. The next day, sections were placed between ACLAR sheets (Electron Microscopy Sciences, Hatfield, USA) and left in the oven at 55°C for 3 days. Regions of interest, from the dentate gyrus, were then excised from the embedded sections with a blade and glued onto resin blocks. Ultrathin (70-80 nm-thick) sections were cut with an ultramicrotome (Leica Ultracut UC7) and collected on square-mesh copper grids prior to imaging at 80 KV with the transmission electron microscope (FEI Tecnai Spirit G2).

**TNF-α and IL-6 ELISA**

Mice were deeply anesthetized with sodium pentobarbital (100 mg/kg) and sacrificed by exsanguination via the inferior vena cava and decapitation. Following collection, blood was centrifuged at 2000 x g for 20 min at 4 °C in heparinized tubes for plasma separation. Plasma was kept at -80°C until analysis. In one group of mice, the whole brain was removed and frozen at -80°C. In another group, the hippocampus was dissected and then frozen at -80°C. Brain or hippocampal homogenates were prepared as described by Qin and Crews [3]. Briefly, frozen brain and hippocampal tissues were homogenized in cold lysis buffer pH 7.4 consisting of 20 mM Tris, 0.25 M sucrose, 2 mM EDTA, 10 mM EGTA, 1% Triton X-100 and a protease inhibitor cocktail tablet (Roche, Indianapolis, USA). Homogenates were centrifuged at 100 000 x g for 40 min at 4°C and supernatants were collected and kept at -80°C until the day of experiment. TNF-α and IL-6 were assessed by ELISA (Invitrogen-Thermo Fisher Scientific, Burlington, Canada) in brain and hippocampal homogenates and plasma samples, following manufacturer's instructions. In order to combine data measured from separate kits, results were expressed as fold change with respect to the control group.

**References**

1. Sadekova N, Iulita MF, Vallerand D, Muhire G, Bourmoum M, Claing A, Girouard H: **Arterial stiffness induced by carotid calcification leads to cerebral gliosis mediated by oxidative stress.** *J Hypertens* 2017.

2. Bisht K, El Hajj H, Savage JC, Sanchez MG, Tremblay ME: **Correlative Light and Electron Microscopy to Study Microglial Interactions with beta-Amyloid Plaques.** *J Vis Exp* 2016.

3. Qin L, He J, Hanes RN, Pluzarev O, Hong JS, Crews FT: **Increased systemic and brain cytokine production and neuroinflammation by endotoxin following ethanol treatment.** *J Neuroinflammation* 2008, **5:**10.
