# Supplementary figures and images for: Differential effect of angiotensin II and blood pressure on hippocampal inflammation in mice
Source: J Neuroinflammation. 2018 Feb 28;15:62. doi: 10.1186/s12974-018-1090-z (PMC6389185; doi:10.1186/s12974-018-1090-z)

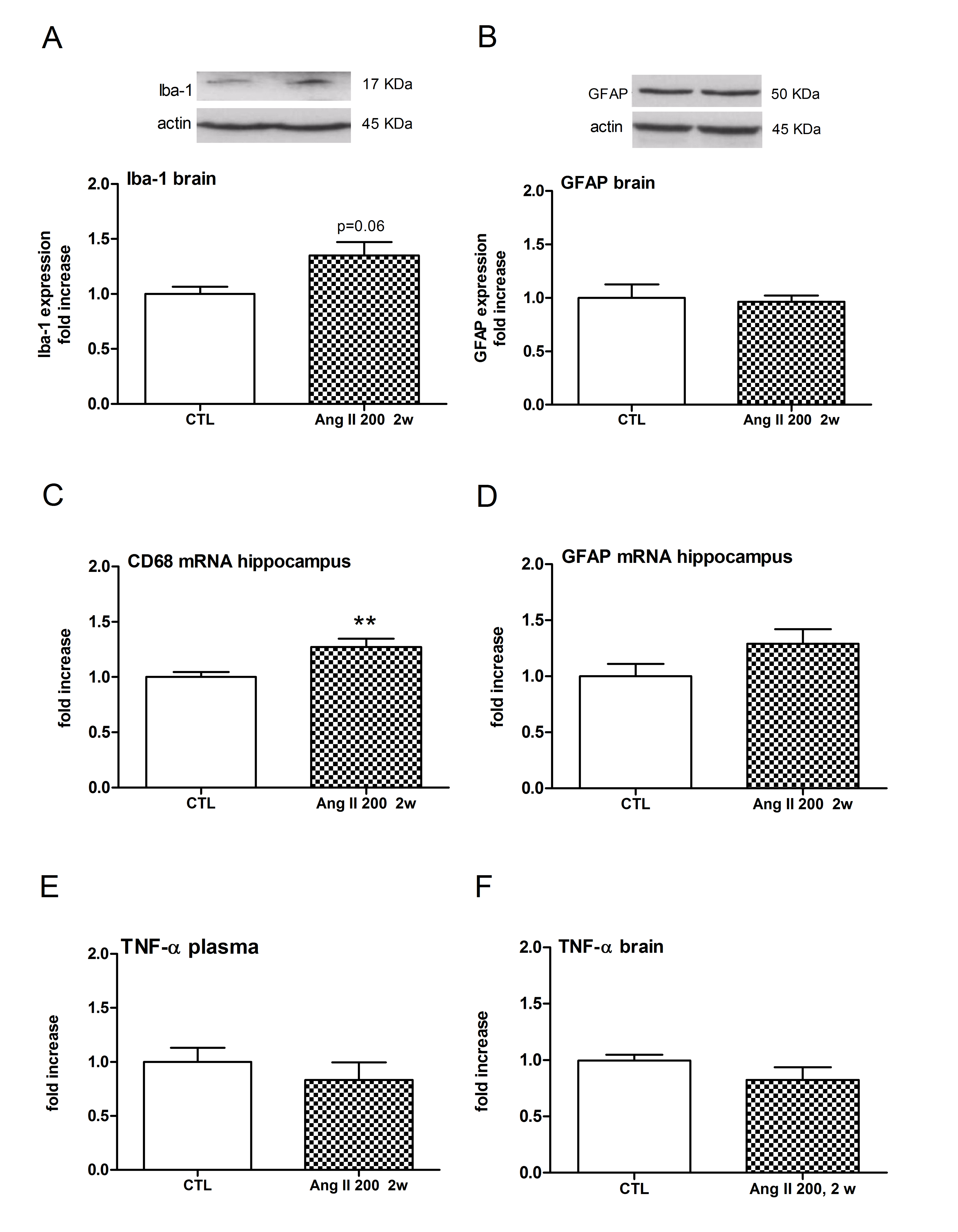

Supplement: Supplementary file 2 — Figure S1.. Effect of extended perfusion of subpressive Ang II on cerebral inflammation. Iba-1 (A) and GFAP (B) were examined by Western blotting in cerebral homogenates after 2 weeks systemic perfusion of Ang II 200 ng/kg/min or 0.9% saline (CTL). Densitometry values of Iba-1 and GFAP were normalized to actin, and the results are expressed relative to the control group. Representative immunoblots are shown (two-tailed Student’s t test, n = 3). CD68 mRNA (C) and GFAP mRNA (D) gene expression were analyzed by qRT-PCR in microdissected hippocampi after 2 weeks systemic perfusion of Ang II 200 ng/kg/min or 0.9% saline (CTL). In each experiment, a treated-to-control ratio was calculated (**p < 0.01, Ang II 200 versus control, by two-tailed Student’s t test, n = 7). ELISA analysis of TNF-α in plasma (E) and in whole brain homogenates (F) after 2 weeks perfusion of Ang II 200 ng/kg/min or 0.9% saline (CTL). A treated-to-control ratio was calculated (two-tailed Student’s t test, n = 6–8). (TIFF 1442 kb) [file 12974_2018_1090_MOESM2_ESM.tif]
